# Genomic Methylation Inhibits Expression of Hepatitis B Virus Envelope Protein in Transgenic Mice: A Non-Infectious Mouse Model to Study Silencing of HBV Surface Antigen Genes

**DOI:** 10.1371/journal.pone.0146099

**Published:** 2015-12-30

**Authors:** Franziska Graumann, Yuri Churin, Annette Tschuschner, Kurt Reifenberg, Dieter Glebe, Martin Roderfeld, Elke Roeb

**Affiliations:** 1 Department of Gastroenterology, Justus Liebig University, Giessen, Germany; 2 Central Laboratory Animal Facility, Johannes Gutenberg University, Mainz, Germany; 3 Institute of Medical Virology, National Reference Centre for Hepatitis B and D Viruses, Justus Liebig University, Giessen, Germany; University of Cincinnati College of Medicine, UNITED STATES

## Abstract

**Objective:**

The Hepatitis B virus genome persists in the nucleus of virus infected hepatocytes where it serves as template for viral mRNA synthesis. Epigenetic modifications, including methylation of the CpG islands contribute to the regulation of viral gene expression. The present study investigates the effects of spontaneous age dependent loss of hepatitis B surface protein- (HBs) expression due to HBV-genome specific methylation as well as its proximate positive effects in HBs transgenic mice.

**Methods:**

Liver and serum of HBs transgenic mice aged 5–33 weeks were analyzed by Western blot, immunohistochemistry, serum analysis, PCR, and qRT-PCR.

**Results:**

From the third month of age hepatic loss of HBs was observed in 20% of transgenic mice. The size of HBs-free area and the relative number of animals with these effects increased with age and struck about 55% of animals aged 33 weeks. Loss of HBs-expression was strongly correlated with amelioration of serum parameters ALT and AST. In addition lower HBs-expression went on with decreased ER-stress. The loss of surface protein expression started on transcriptional level and appeared to be regulated epigenetically by DNA methylation. The amount of the HBs-expression correlated negatively with methylation of HBV DNA in the mouse genome.

**Conclusions:**

Our data suggest that methylation of specific CpG sites controls gene expression even in HBs-transgenic mice with truncated HBV genome. More important, the loss of HBs expression and intracellular aggregation ameliorated cell stress and liver integrity. Thus, targeted modulation of HBs expression may offer new therapeutic approaches. Furthermore, HBs-transgenic mice depict a non-infectious mouse model to study one possible mechanism of HBs gene silencing by hypermethylation.

## Introduction

Infection with the hepatitis B virus (HBV) is a serious global health problem. Worldwide, more than 240 million people are currently chronic HBV carriers and 25% of the chronic HBV-infected patients develop severe liver diseases [1], like liver cirrhosis and hepatocellular carcinoma (HCC) [2]. Thus HBV infection is one of the most prevalent infectious diseases worldwide [3].

HBV is a hepatotropic virus which belongs to the hepadnaviridae family [4]. It is widely accepted that liver damage is mainly caused by interactions between HBV and the immune system [5–7]. Although replication of wildtype HBV is not regarded as being cytopathic, several studies showed that HBV infection in some cases may cause direct liver damage by intracellular accumulation of HBs and subsequently induced ER-stress [5,8,9]. In this regard, model systems like HBs transgenic mice have been used to elucidate underlying mechanisms [5]. Transgenic mice expressing the large HBs protein (LHBs) reflect the situation in the liver of immune-suppressed HBV-infected patients demonstrated strong retention of HBs in hepatocytes especially in late phases of chronic HBV infection [5].

Recently, epigenetic mechanisms, including methylation of viral DNA have been shown to control the production of viral proteins [10,11]. The methylation of distinct CpG sites in the HBV genome was shown to be associated with HCC [12]. Furthermore, the silencing of HBs genes by methylation was suggested to result in occult HBV cases [12]. Recent studies demonstrated that CpG island 2 may be most relevant to the regulation of the surface gene [13].

ER stress triggers a specific cellular response known as unfolded protein response (UPR). After initial activation of UPR via chaperone GRP-78 three mechanisms are used to restore ER-homeostasis: 1) Upregulation of protein expression promoting the protein folding. 2) Global inhibition of mRNA translation to minimize the amount of produced proteins. 3) Induction of apoptosis if UPR is not sufficient to compensate ER-stress [14,15].

Distinct branches of UPR are mediated by three different classes of ER-membrane transducers: inositol requiring protein-1 (IRE1), activating transcription factor-6 (ATF6) or protein kinase-like endoplasmic reticulum kinase (PERK). Expression of apoptosis-associated nuclear factor CHOP is mediated by phosphorylation of eIF2α [16] that in turn is phosphorylated by PERK [17].

UPR ameliorates the accumulation of unfolded proteins in the ER, but can also induce cell death if these events are severe or protracted. Transgenic mice used in the present study show an overexpression of the large and small hepatitis B virus surface proteins (LHBs and SHBs, respectively) [18], which effectuates blocking of subviral (SVP) and virion particles secretion within the secretory pathway [9]. The consequent accumulation of LHBs leads to direct cell damage by induction of ER-stress [9,18–21]. Activation of ER stress genes as a consequence of intracellular HBs accumulation is thought to be involved in hepatocarcinogenesis [8,22]. Therefore, therapeutic reduction of intracellular HBs accumulation and subsequent ER stress might be a promising tool to treat patients with chronic hepatitis B.

HBs-transgenic hybrids of the inbred mouse strains C57BL/6-J and SJL-J developed HBs negative nodules and HCC with ongoing liver disease [23]. The emerging HCCs were HBs-negative like regenerative nodules. However, a correlation between these nodules and HCC-development was disproved. A more recent study demonstrated that HCCs grew out of hepatocytes with intact transgene and that regenerative nodules were produced by HBs-negative progenitor cells [24].

In the present study, spontaneous loss of the HBs expression in transgenic mice was shown to be associated with HBV genome methylation.

## Materials and Methods

### Animal Model

Handling and holding conditions were described before [5]. Transgenic mice were maintained at the Central Animal Laboratory of the Justus-Liebig-University Giessen under specified pathogen-free conditions. This study was carried out in strict accordance with the recommendations in the Guide for the Care and Use of Laboratory Animals of the German law of animal welfare. The mice received humane care, and all experiments were approved by the Committee on the ethics of Animal Experiments of the Regierungspraesidium Giessen, Giessen, Germany (permit number: V54-19c 20 15 h 01 GI20/10 No. 52/2011 and No. 48/2012). All efforts were made to minimize suffering.

Mice were analysed for the presence of antibodies to several mouse pathogens known to be associated with liver disease, including mouse hepatitis virus, Sendai virus, ectromelia virus, and lymphocytic choriomeningitis virus, by mfd Diagnostics (Wendelsheim, Germany).

Generation and characteristics of transgenic lineage C.B6J-Tg(Alb1HBV)44Bri (HBVTg/c) has been described previously [5,23]. Transgenic mice expressed HBs under control of the mouse Alb promotor. At age of 5, 12, 19, 26, and 33 weeks mice were anaesthetized by Isofluran inhalation and subsequently killed by cervical dislocation. Liver samples were collected and preserved for analyses. Serum samples were stored at -80°C until analysis of alanine-aminotransferase (ALT), aspartate-aminotransferase (AST) and alkaline phosphatase (AP) by routine clinical chemistry on a Reflotron Plus Analyzer (Roche, Mannheim, Germany).

### Histology

Histology was performed as described before [5]. Briefly, immediately after necropsy, liver samples for histology were fixed in 1% neutral buffered paraformaldehyde at 4°C for 16 hours and embedded in paraffin. Paraffin sections (5μm) were stained with hematoxylin and eosin (HE) or 0.1% Sirius Red F3B in saturated picric acid (Chroma, Münster, Germany) for the detection of collagen fibers [25].

### Immunohistochemistry

Immunohistochemistry was performed as described before [5]. Immunohistochemistry (IHC) was performed using ImmPRESS Peroxidase Detection Reagents (Vector Laboratories) and antibodies specific for HBs (20-HR20, Fitzgerald), GADD153 (F-168, Santa Cruz, Cytokeratin 19 (M-17, Santa Cruz), LHB [26], ATF-3 (C19, Santa Cruz). Colour reaction was developed with VECTOR VIP Peroxidase Substrate Kit or DAB Peroxidase Substrate Kit, (Vector Laboratories).

### Western blot Analysis

Protein samples were prepared from total liver lysates. Protein samples were boiled for 5 min, chilled on ice, and subjected to 12% SDS- PAGE and transferred to polyvinylidene difluoride membranes. Visualization of proteins was performed by Horseradish-Peroxidase (HRP)-linked antibodies. ECL Chemiluminescence Detection Kit (SuperSignal West Pico Chemiluminescent Substrate, Thermo Scientific, Darmstadt, Germany) was used according to the manufacturer’s protocol. BioDoc Analyze 2.1 (Biometra, Göttingen, Germany) was used for semiquantitative analysis (S1 Table).

### HBs ELISA

HBs was measured in liver lysates by a sandwich ELISA as described before [27].

### mRNA-Isolation

To isolate mRNA from liver tissue we used RNeasy-Mini-Kit according to the manufacturer´s protocol (Qiagen, Hilden, Germany). The mRNA was reverse transcribed to cDNA with iScript cDNA Synthesis Kit according to the manufacturer´s protocol (Bio-Rad, Munich, Germany).

### Isolation of genomic DNA

For isolation of genomic DNA from liver tissue we used Pure Link^TM^ Genomic DNA Mini Kit according to the manufacturer´s protocol (InvitroGen, Karlsruhe, Germany).

### Quantitative Real-time-PCR

For quantification of HBs-mRNA and genomic DNA qRT-PCR was used (Primer and PCR characteristics: S1 Table). We used r18S as housekeeping gene control.

### DNA Methylation

Genomic DNA from frozen liver samples was isolated using the Proteinase K/phenol method [28]. Isolated genomic DNAs were modified with the EpiTect Plus DNA Bisulfite Kit, according to the manufacturer´s protocol (Qiagen). Amplification of two CpG island regions from HBV fragment was performed using EpiTaq HS polymerase (Clontech) and the following primer pairs: CpG island I,

5′- TTTTTATTAAATTTTGTAAGATTTTAGAGTGAGAGG -3′ and 5′- TAAAAACTACRAATTTTAACCAAAACACAC -3′; CpG island II, 5′- AATATATATYGTTTTTATGGTTGTTAGGTTGTG -3′ and 5′- AAAATCCAAAAATCCTCT TATATAAAACCTTAAAC -3′.

The actual methylation status of amplified fragments was determined through direct PCR product sequencing.

### Statistical analysis

Statistical analysis was performed with SPSS V. 22.0 software (SPSS Inc.). For non-normally distributed parameters, Mann-Whitney U test and Spearman rank test were applied. Box- and Whisker Plots show median and interquartile range with 95% confidence interval. The upper and lower hinges of the box represent the 75th and 25th percentile, respectively. P values <0.05 were considered statistically significant.

## Results

### Loss of HBs expression is age dependent

**Fig 1:**
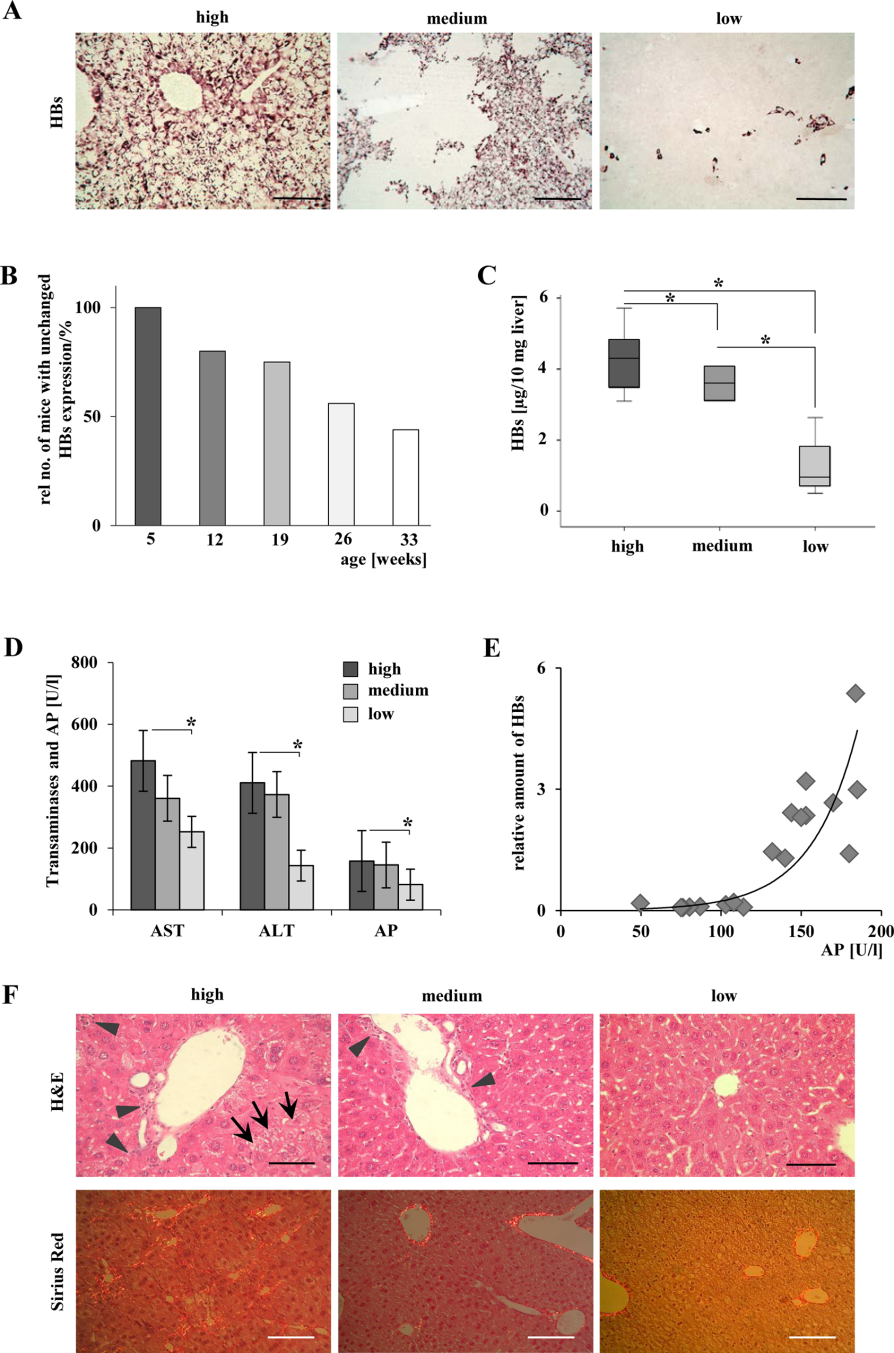
Loss of HBs expression ameliorated liver injury. (A) Immunohistochemical staining of HBs demonstrated the appearance of hepatic areas without transgene expression in distinct mice beginning with the age of 12 weeks. At the same age, mice with HBs expression in single hepatocytes and mice with expression of HBs in every hepatocyte were observed. Representative micrographs are shown. Scale bars in left and right panels: 160μm, magnification x200. Scale bar 320 μm in the medium panel: magnification x100. (B) The observed loss of HBs expression is age-dependent. The relative number of mice exhibiting areas without HBs expression increased with higher age of the mice. Bars indicate the relative number (%) of animals with unchanged HBs expression in all hepatocytes. (C) Quantitative analysis of HBs expression in liver tissue assessed by ELISA. *P < 0.05. (D) Amelioration of liver serum parameters correlates significantly with loss of HBs expression. *P < 0.05. (E) Correlation of AP and HBs. r^2^ = 0.864, P < 0.001. (F) H&E staining indicates ground glass hepatocytes and enhanced numbers of inflammatory cells in the „high”and „medium”group. Scale bars 80 μm, magnification x400. Arrowheads: inflammatory infiltrates, arrows: ground-glass-hepatocytes. Sirius Red staining indicates fibrillary collagen. Scale bars 160μm, magnification x200.

**Fig 2:**
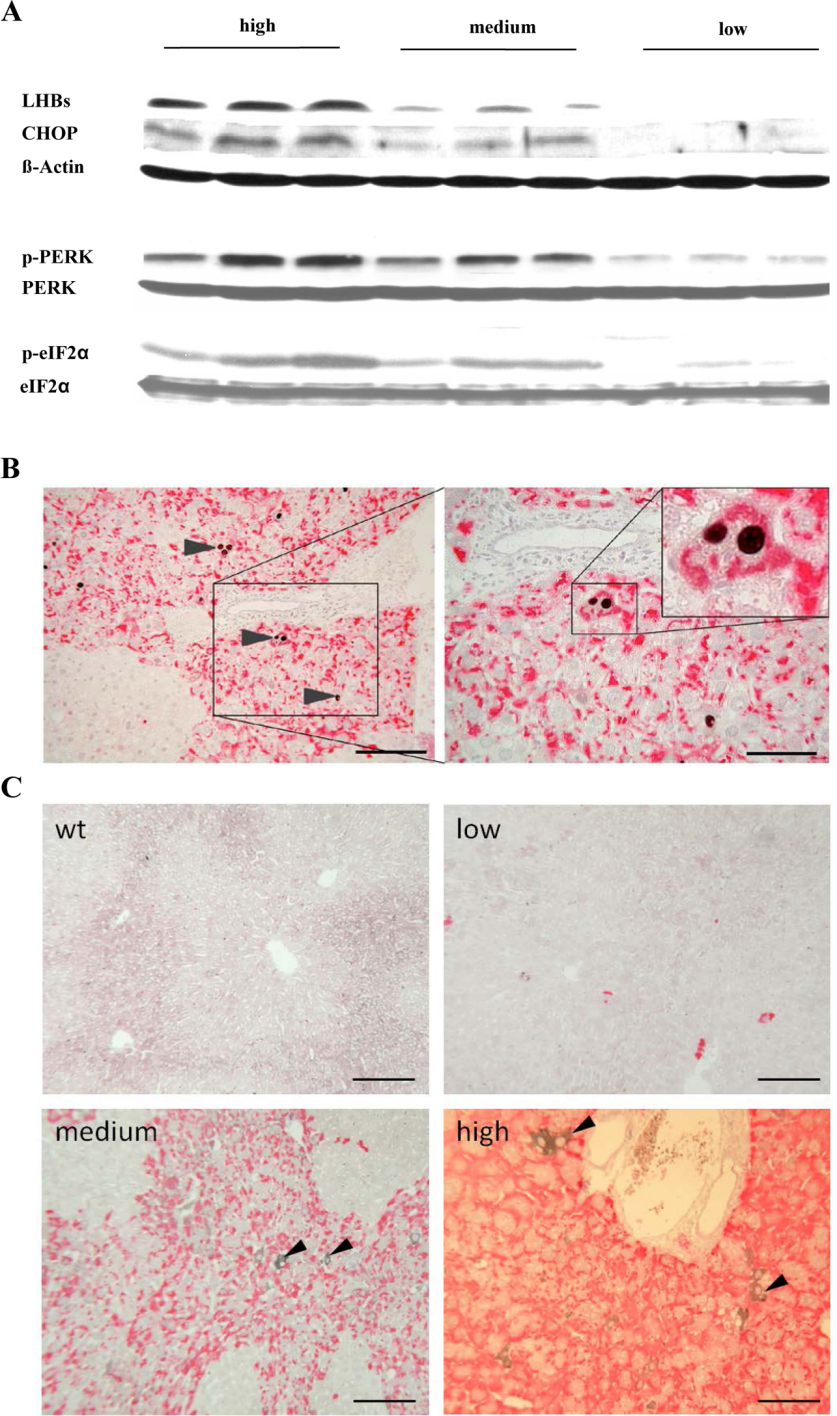
Spontaneous loss of HBs expression reduced ER-stress and apoptosis. A) Representative Western blots of p-eIF2α, p-PERK, and CHOP demonstrated the correlation between HBs expression and activation of ER-stress. Loading controls: ß-Actin, eIF2α, and PERK. (B) Co-immunostaining of HBs (red) and CHOP (black) in transgenic mouse liver. The micrographs demonstrate that HBs expressing hepatocytes undergo apoptosis only. Scale bars 160/80 μm. Arrowheads indicate CHOP stained apoptotic hepatocytes. (C) Co-immunostaining of HBs (red) and GRP78 (black) in transgenic mouse liver. IHC demonstrated strong expression of GRP78 in selected hepatocytes in centrilobular areas. Scale bars 50 μm. Arrowheads indicate GRP78 stained hepatocytes.

Transgenic mice of both gender were sacrificed at week 5, 12, 19, 26, and 33 (n = 8–10 per group). All mice included were identified to be transgenic for HBs by genotyping. The spontaneous reduction in expression of HBs was observed in immunohistochemically stained liver samples of some elder mice (Fig 1A). HBs free areas appeared irregular in shape. The effect appeared in 20% of mice aged 12 weeks. The relative number of mice with reduced HBs-expression was 45% at the age of 26 weeks and 56% in 33 weeks old mice. Younger mice at the age of 5 weeks did not show any loss of HBs expression (Fig 1B). In order to define mouse groups with reduced transgene expression, 19–33 weeks old mice were split into three groups: high (n = 9, all hepatocytes were stained positive for HBs), medium (n = 5, 11–99% of all hepatocytes were stained positive for HBs), and low (n = 4, ≤10% of hepatocytes were stained positive for HBs) expression of HBs protein (Fig 1A). Significant differences in HBs expression were validated by ELISA and Western blotting (Figs 1C and 2A). Quantitative ELISA data revealed significant differences in HBs protein expression between all three groups. This clearly defines each group and furthermore underlines the distinguished character of the ´high´, ´median´, and ´low´ group. No gender specific effects were observed in this model.

Aminotransferases and alkaline phosphatase were measured to estimate liver cell damage. Integrity of liver parenchyma was correlated with the amount of HBs (Fig 1D). A significant amelioration of hepatic serum parameters was found in the group of mice with low HBs expression (p(AST) = 0.016; p(ALT) = 0.031; p(AP) = 0.005). In addition, decreased AP-levels showed the strongest correlation with the amount of HBs expression (r^2^ = 0.864; p<0.001) (Fig 1E). With decreasing HBs expression less inflammatory cells infiltrated periportal fields (Fig 1F). Sirius Red staining demonstrated reduced fibrosis in correlation with loss of HBs (Fig 1F).

### Loss of HBs expression reduces ER-stress

In order to define the underlying mechanism for amelioration of liver damage by loss of HBs, ER-stress and unfolded protein response were analysed. As shown recently, expression of HBV surface proteins in the liver of transgenic mice on BALB/c genetic background induced activation of PERK, eIF2α, glucose regulated protein (GRP) 78, and pro-apoptotic C/EBP homologous protein (CHOP) [5]. For this reason the activation of these proteins was analysed by Western blotting. Similar levels of CHOP, PERK, and eIF2α activation were detected in the liver of mice within the same HBsAg expression level group. Interestingly, Western blot analysis showed a correlation between the amount of HBs accumulation and the activation of p-PERK, p-eIF2α, and CHOP (Fig 2A). The activation of these proteins peaked with the highest expression of HBs and dropped with reduction of HBs. Taken together, these data provide evidence that HBs specific activation of the PERK branch of UPR is not static but reversibly and dynamically associated with the HBs expression level.

To examine the location of CHOP expressing hepatocytes immunohistochemistry (IHC) was performed. A significant part of hepatocytes expressing HBs accumulated CHOP in the nucleus, whereas no nuclear translocation of CHOP was observed in HBs free areas (Fig 2B). Thus, co-staining for HBs and CHOP demonstrated hepatocyte apoptosis in HBs-expressing areas only (Fig 2B). Additionally, IHC demonstrated strong expression of GRP78 in selected hepatocytes in centrilobular areas (Fig 2C). Furthermore, expression of GRP78 was found in selected hepatocytes that appeared exclusively in HBs positive areas but not in HBs free areas.

### Loss of HBs-expression is caused by DNA methylation

**Fig 3:**
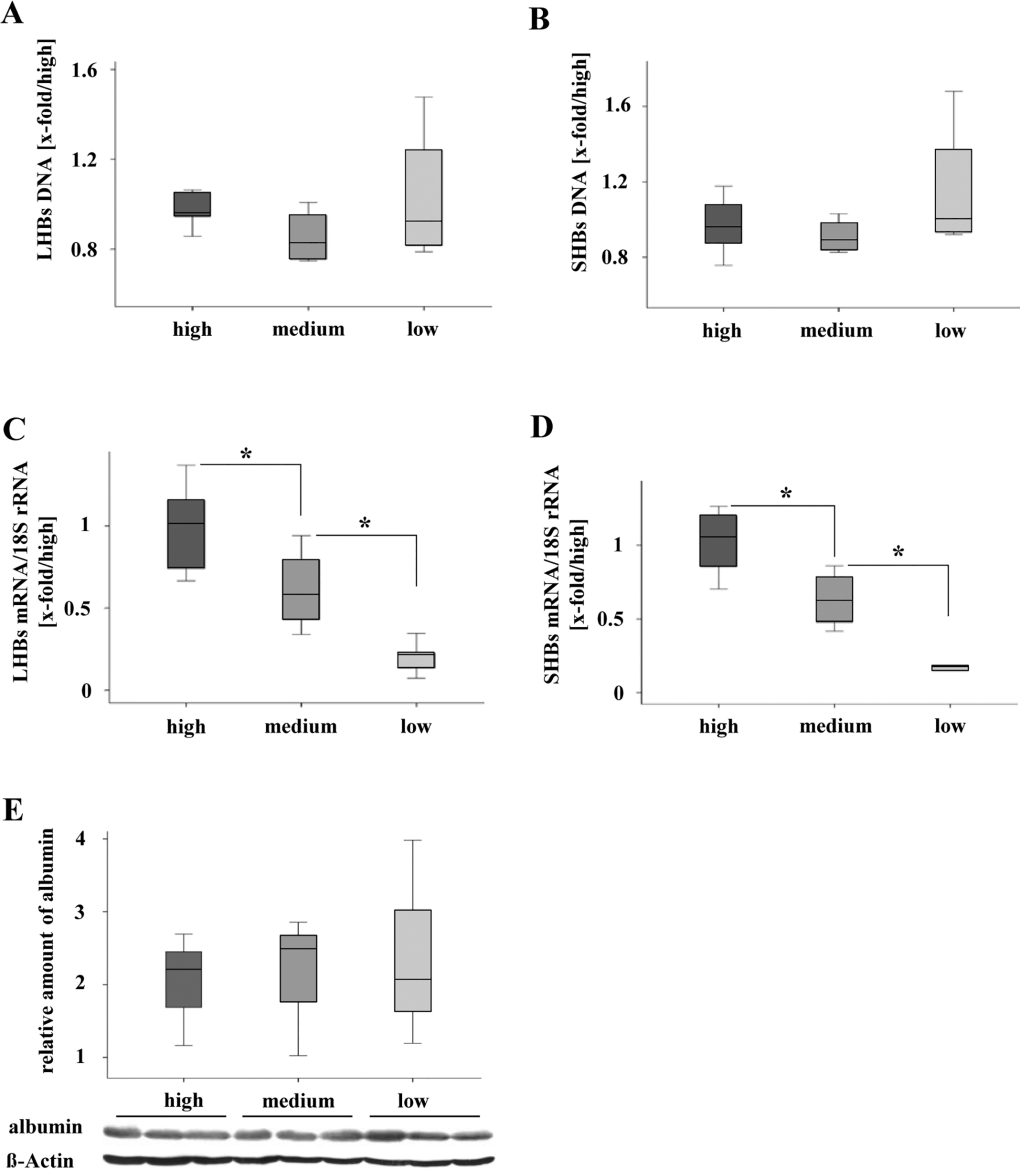
Analysis of transgenome integrity and transcription of HBs. (A) and (B) qRT-PCR-analysis demonstrated stable transgenome DNA (LHBs, SHBs) in liver tissue with different amounts of HBs protein expression. Normalized to ´high´ group. (C) and (D) Significant difference of mRNA-levels (LHBs, SHBs) were shown by qRT-PCR-Analysis. Normalized to ´high´group and 18S rRNA. *P < 0.05. (E) Western blot analysis reveals equal accumulation of albumin protein among the three groups with different HBs expression.

In order to define the reason for loss of HBs expression, DNA, mRNA, and the function of the albumin promoter were analysed. qRT-PCR for LHBs- and SHBs- genomic DNA (integrated into the mouse genome) did not show any significant differences between all three experimental groups (Fig 3A and 3B). Accumulation of HBs mRNA of the low- (LHB and SHB: p = 0.006) and the medium group (LHB: p = 0.027; SHB: p = 0.014) was decreased significantly (Fig 3C and 3D). Western blot analysis revealed the same level of albumin protein expression in all three experimental groups (Fig 3E).

**Fig 4:**
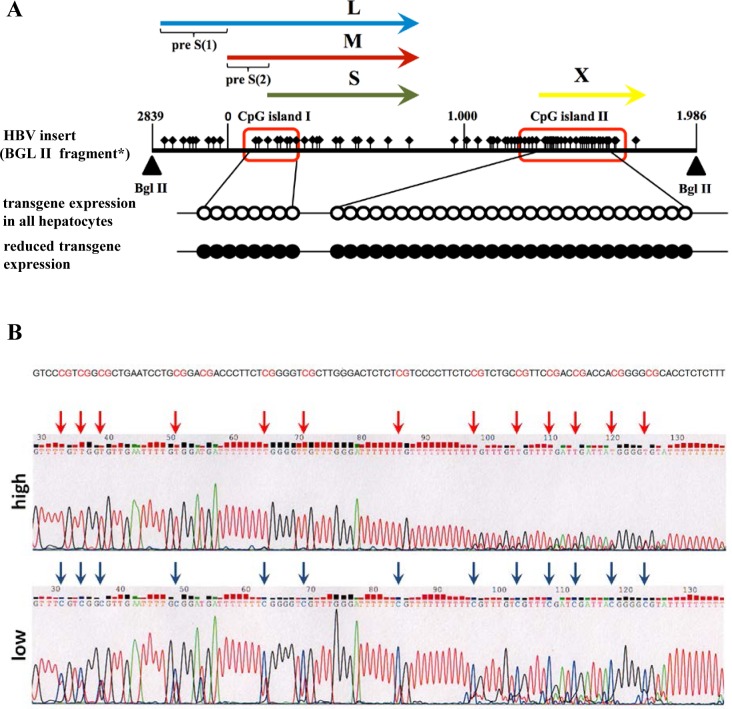
Methylation analysis of HBV-genomic sequence. A) Mice with reduced amount of transgene expression exhibited methylated CpG islands I+II (red boxes, figure modified from [12]). Unfilled circles indicate non-methylated CpG sites. Presence of methylated CpGs is indicated by black filled circles and was detected by analyzing six independent mice with reduced transgene expression. L, M, and S indicate the sequences encoding the large, middle, and small surface proteins. X indicates the sequences encoding the X-protein. *HBV insert (Bgl II fragment) according to Chisari et al. 1986 [30]. B) A representative result of Bisulfite sequenced CpG island II demonstrates methylation of all CpG sites in mice with reduced HBs expression (low).

It has been reported that DNA methylation of HBV in human cells may undergo dynamic changes [29] and methylation regulated viral protein production [13]. Herein, the HBV DNA fragment integrated in mouse genome contains two CpG islands I and II [12]. Bisulphite sequencing of the amplified products of CpG island I, and II revealed their hypermethylation in mice with reduced HBs expression (Fig 4). Thus, hypermethylation of HBV DNA in mouse genome correlated with reduced HBs expression.

### Loss of HBs expression reduced ductular response

**Fig 5:**
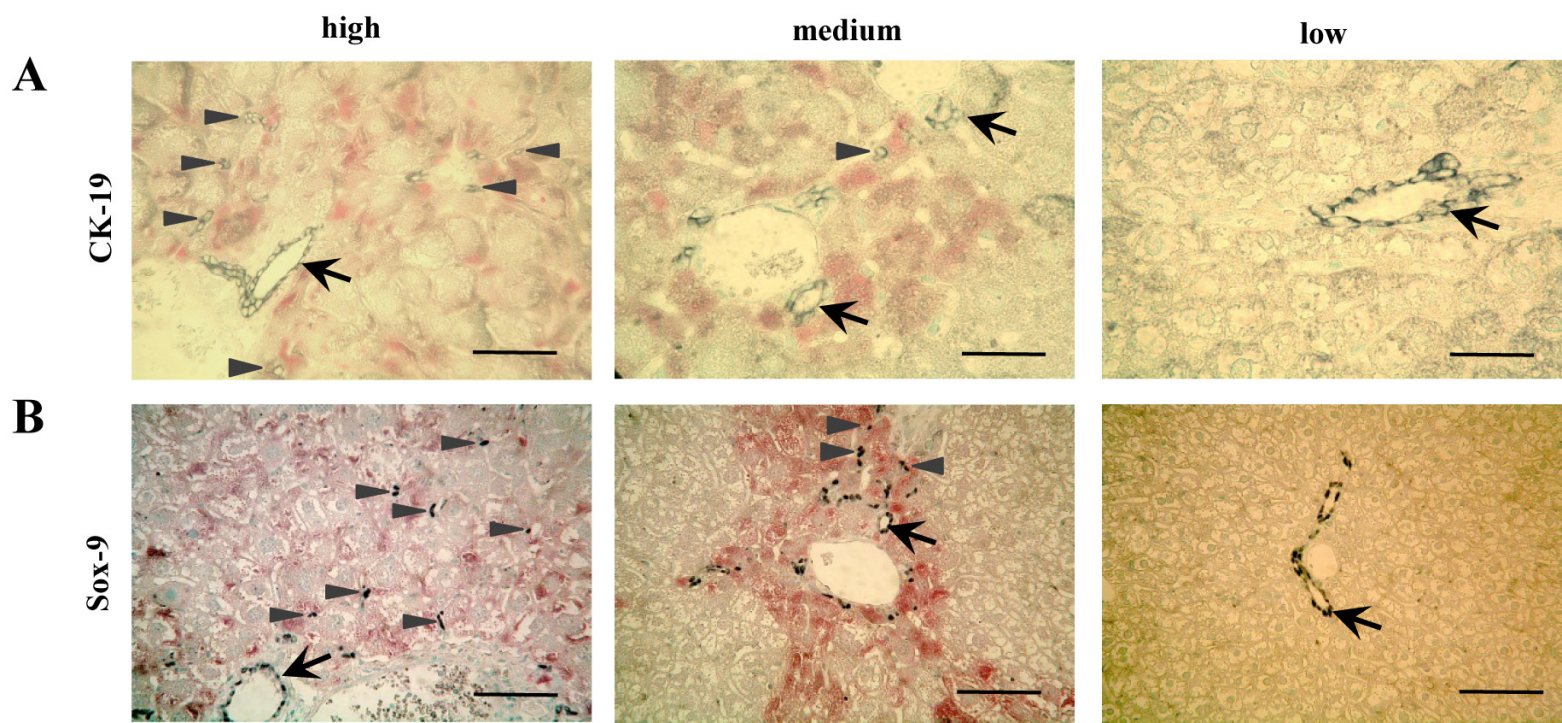
Loss of HBs-Expression reduced regeneration. (A) Immunohistochemical staining of CK-19 (grey) shows that HBs expression (red) in every hepatocyte went along with higher numbers of single CK-19^+^-oval cells around periportal fields. Scale bar 32 μm. Arrow: CK-19 positive bile duct epithelium, arrowheads: single CK-19 positive cells in parenchyma. (B) Immunohistochemical staining of Sox-9 (black) indicates that areas with HBs expression (red) exhibited more Sox-9^+^-oval cells in periportal tracts and surrounding parenchyma than areas without HBs expression. Scale bar 80 μm. Arrow: Sox-9 positive cells around a bile duct, arrowhead: Sox-9 positive cells in parenchyma.

Cytokeratin-19 (CK19) [31] and Sox-9 [32] were stained to detect regenerative oval cells. Fig 5 demonstrates lower numbers of CK-19 and Sox-9 positive cells infiltrating parenchyma around portal fields in mouse liver with reduced HBs expression. Please note that CK-19 and Sox-9 positive cells infiltrate parenchyma in HBs expressing areas (red staining) but not those areas with loss of HBs expression.

## Discussion

In order to find the mechanistic basis for the reduction of transgene expression we proofed genomic integrity of the transgene and integrity of the transgene promotor Alb. Furthermore, loss of mRNA accumulation and epigenetic modifications of the integrated part of the HBV-genome was shown. Interestingly, the transgenome specific hypermethylation of specific CpG islands correlates with loss of HBs expression and thus is suggested to be the cause of the sharp decrease of HBs production. Our data imply functional consequences of this specific and likely transient hypermethylation and thus strengthen prior studies demonstrating that DNA methylation of the HBV genome acts as a part of the host surveillance machinery to modulate the production of viral proteins [12].

Concomitantly to observations in woodchucks and chimpanzees [33,34] a more recent study demonstrated that non-neoplastic clonal expansion of normal-appearing hepatocytes exists in tissue samples of patients chronically infected with HBV [24]. The latter study determined extensive clonal expansion of hepatocytes in human HBV carriers, particularly in the non-cirrhotic liver. Furthermore, it was shown that clonal expansion included normal-appearing hepatocytes, but not neoplastic hepatocytes [24]. It was speculated that immune killing may exert selective pressure on the population of infected hepatocytes, leading it to evolve in order to survive [24]. The shape and pattern of clonally expanding HBs-free hepatocytes (Fig 1) appeared normal without cellular dysplasia, and compression of surrounding tissue that has been shown in HBs-free spherical hepatocellular neoplasms from HBs tg mice on genetic background C57BL6-J [23].

It has been shown before that liver injury in HBs transgenic mice increased in age-dependent manner with higher intrahepatic concentrations of HBs [18]. Accordingly, herein it is demonstrated that amelioration of established liver injury is also possible with spontaneous loss of hepatic HBs proteins (Fig 1). Moreover, also the underlying mechanisms of ER stress and subsequent induction of apoptosis vanished with spontaneous loss of intracellular HBs accumulation (Fig 2). Interestingly, the activation of this branch of ER-stress mediated apoptosis was restricted to hepatocytes demonstrating intracellular accumulation of HBs (Fig 2B). Thus, the spontaneous loss of surface protein expression protected animals from progressive liver disease by reduction of hepatocellular stress.

Being exposed to permanent damage, liver regeneration by compensatory hyperplasia of hepatocytes seems to be insufficient. In this case oval cell might be activated [8], which proliferate and differentiate near the periportal areas and subsequently migrate into the parenchyma [35,36]. CK-19 or Sox-9 positive stem cells enter the parenchyma from the portal tract in oval cell associated regeneration [31,32]. In the current model is has been shown that single CK-19 or Sox-9 positive stem cells exclusively appeared in areas with high HBs expression (Fig 5). This observation is in line with the ameliorative effects that were directly caused by loss of HBs expression. Regeneration by stem cells became needless in healthy parenchyma without perturbation by the noxa causing chronic liver injury.

The relatively small number of animals used for this study originates from the fact, that the described effect was randomly noticed in elder mice mainly. Nevertheless, the number of mice was definitely sufficient for statistical evaluations.

In summary, the current study demonstrates that

1. HBs-transgenic mice on BALB/c-genetic background lost transgene expression spontaneously and age dependently likely mediated by methylation of a specific site of HBV DNA.
2. Loss of HBs-expression had protective effects on the liver and correlates with reduced ER-stress.

In conclusion, HBs transgenic mice on BALB/c background represent a suitable and non-infectious model to study one possible mechanism of silencing of HBs expression by methylation. Ameliorated cell stress and liver integrity after loss of HBs expression demonstrate the therapeutic potential of targeted modulation of HBs expression.

## Supporting Information

S1 Table Primer and PCR conditions for qRT-PCR. HBs-mRNA and genomic DNA qRT-PCR was analysed by qRT-PCR using primer and PCR characteristics presented in STab 1. We used r18S as housekeeping gene control. AT = annealing temperature, bp = base pairs, LHB = large HBV surface protein, SHB = short HBV surface protein.
(DOCX)
