# Supplementary material for: Genomic Methylation Inhibits Expression of Hepatitis B Virus Envelope Protein in Transgenic Mice: A Non-Infectious Mouse Model to Study Silencing of HBV Surface Antigen Genes
Source: PLoS One. 2015 Dec 30;10(12):e0146099. doi: 10.1371/journal.pone.0146099 (PMC4696744; doi:10.1371/journal.pone.0146099)
Supplement: S1 Table — HBs-mRNA and genomic DNA qRT-PCR was analysed by qRT-PCR using primer and PCR characteristics presented in STab 1. We used r18S as housekeeping gene control. AT = annealing temperature, bp = base pairs, LHB = large HBV surface protein, SHB = short HBV surface protein. (DOCX) [file pone.0146099.s001.docx]

**S1 Table: Primer and PCR conditions for qRT-PCR**

|  | AT/°C | length (bp) | Primer sequences |  |
| --- | --- | --- | --- | --- |
|  |  |  | sense | antisense |
| r18S | 57 | 22 | 5'- gat cag ata ccg tcg tag ttc c-3' | 5'- tat caa tct gtc aat cct gtc c-3' |
| SHB | 57 | 20 | 5´-ccc aac ctc caa tca ctc ac-3´ | 5´-gca gca gga tga aga gga ag-3´ |
| LHB | 57 | 20 | 5´-gtt gga tcc agc ctt cag ag-3´ | 5´-tcc agc tcc tac ctt gtt gg-3´ |

HBs-mRNA and genomic DNA qRT-PCR was analysed by qRT-PCR using primer and PCR characteristics presented in STab 1. We used r18S as housekeeping gene control. AT=annealing temperature, bp= base pairs, LHB=large HBV surface protein, SHB=short HBV surface protein
